# EBRA Migration Analysis of a Modular, Distally Fixed Stem in Hip Revision Arthroplasty: A Clinical and Radiological Study

**DOI:** 10.3390/jcm11195857

**Published:** 2022-10-03

**Authors:** Philipp Blum, David Putzer, Johannes Neugebauer, Markus Neubauer, Markus Süß, Dietmar Dammerer

**Affiliations:** 1 Department of Trauma Surgery, BG Trauma Center Murnau, Prof.-Küntscher-Str. 8, 82418 Murnau, Germany; 2 Department of Orthopaedics and Traumatology, Medical University of Innsbruck, Anichstraße 35, 6020 Innsbruck, Austria; 3 Department of Experimental Orthopaedics, Medical University of Innsbruck, Sonnenburgstr. 16, 6020 Innsbruck, Austria; 4 Department for Orthopaedics and Traumatology, University Hospital Krems, Mitterweg 10, 3500 Krems an der Donau, Austria

**Keywords:** total hip arthroplasty, revision, modular reconstruction prosthesis, Einzel-Bild-Röntgen-Analyse, subsidence

## Abstract

Background: Massive osteolysis of the proximal femur makes stem revision a challenging procedure. EBRA-FCA provides the opportunity to determine stem migration, which is considered a predictive factor for implant survival. In this study, we aimed to analyze the migration behavior of a modular, distally fixed reconstruction prosthesis. Methods: Applying a retrospective study design, we reviewed all consecutive patients who received a cementless MP reconstruction prosthesis (Waldemar Link GmbH & Co. KG, Hamburg, Germany) at our Department between 2005 and 2019. We reviewed medical histories and performed radiological measurements using EBRA-FCA software. Results: A total of 67 stems in 62 patients (female 26; male 36) fulfilled our inclusion criteria. Mean age at surgery was 68.0 (range 38.7–88.44) years. EBRA migration analysis showed a median subsidence of 1.6 mm (range 0.0–20.6) at 24 months. The angle between stem and femur axis was 0.3° (range 0.0°–2.9°) at final follow-up. No correlation between body mass index and increased subsidence was found (*p* > 0.05). Overall revision-free rate amounted to 92.5% and revision-free rate for aseptic loosening to 98.5%. Furthermore, no case of material breakage was detected. Conclusions: In summary, the MP reconstruction prosthesis showed low subsidence and reduction in the migration rate over the investigated follow-up. Based on this, the modular stem can be considered as a good therapy option in challenging stem revisions offering various options to address the individual anatomical situation.

## 1. Introduction

Implantation of primary total hip arthroplasty (THA) is considered a successful and cost-effective procedure. The combination of an increasing number of patients undergoing THA and a rising life expectancy can be expected to require more revision surgeries in future [1,2]. Periprosthetic fractures as well as infections and massive osteolysis due to aseptic loosening can lead to pronounced bone loss of the proximal femur, making revision a challenging procedure for the surgeon [3,4]. To achieve implant stability in such bony defects, various fixation options are available. In addition to non-cemented and cemented monoblock components used with or without the bone augmentation technique, there are also patient-specific modular or custom-made implants [3]. In the 1980s, Wagner was the first to describe the principle of a tapered, fluted, titanium implant firmly anchored in the distal and intact part of the femur, which should allow regeneration of the proximal defect [5].

The component investigated in this study is the MP reconstruction prosthesis by Link (Waldemar Link, Hamburg, Germany), which is a modular stem based on Wagner’s principle. According to the Swedish Hip Arthroplasty Register, the MP stem was the most used stem in uncemented revisions in 2019, with a percentage of 37.1 [6].

Aseptic loosening is the most common cause of failure in revision hip arthroplasty [7]. Distal migration of the stem, also called subsidence, was found to be a good predictive factor for aseptic loosening in several previously published studies [8,9,10,11]. In this context, Einzel-Bild-Röntgen-Analyse-Femoral Component Analysis (EBRA-FCA) is a computer-assisted method for measuring the distal migration of femoral stems using standard anterior–posterior (ap) pelvic radiographs without requiring additional means at exposure (e.g., ball markers). It has proven accuracy and a sensitivity of more than 1 mm in detecting migration as compared to roentgen stereophotogrammetric analysis (RSA) [12,13].

The aim of this study was to investigate the clinical outcome and migration behavior of a modular tapered stem using EBRA-FCA in the first 24 months.

## 2. Materials and Methods

The study was approved by the local ethics committee (Medical University of Innsbruck, Austria, Europe). In the present retrospective study, we reviewed all consecutive patients who received an MP stem at our department between 2005 and 2019. During this time, a total of 101 cementless (n = 86) and cemented (n = 15) MP femoral reconstruction prostheses were implanted.

The MP reconstruction prostheses is a modular system, available in a cementless and a cemented version and consists of the following main components: head, neck segment, optional proximal spacer and the stem. The cementless stem has a titanium alloy with a grit blast finished surface, creating a microporous structure of 70 µm to promote bone ingrowth. Next to this, the stem has a 3° kink to follow the natural femoral bow. Distally, longitudinal ribs provide rotational stability and reduce stem stiffness. It is available in 6 lengths (160 mm–330 mm) and 7 diameters (12 mm–25 mm). The cemented stem has a CoCrMo alloy, is anatomically curved and available in 4 lengths (200 mm–320 mm) and 3 diameters (12 mm–16 mm). The body of the modular neck segment has a round cross-section with a microporous surface, is available in the size of 35 mm and 65 mm, with or without collar, standard or “XXL” lateral offset, and with CCD angles of 126 and 135 degrees. The base of the neck segment is toothed so that the anteversion can be set precisely for each patient. Proximal spacers allow the correction of leg length discrepancies between 10 mm and 30 mm and can be inserted between the proximal end of the stem and the neck segment if necessary. Prosthesis heads are available in CoCrMo alloy or aluminum oxide ceramic [14].

The medical histories have been screened for sociodemographic data, surgical approach, body mass index, cut-to-suture time, material breakage and the preoperative diagnosis for THA indication. In the case of a periprosthetic femoral fracture, it was categorized using the Vancouver classification. Next to this, range of motion was recorded preoperatively and up to one year after surgery by surgeons at our department using a goniometer during clinical examination. In addition, the latest anterior–posterior (ap) X-ray was analyzed for radiolucent regions according to the Gruen zones [15].

**Figure 1:**
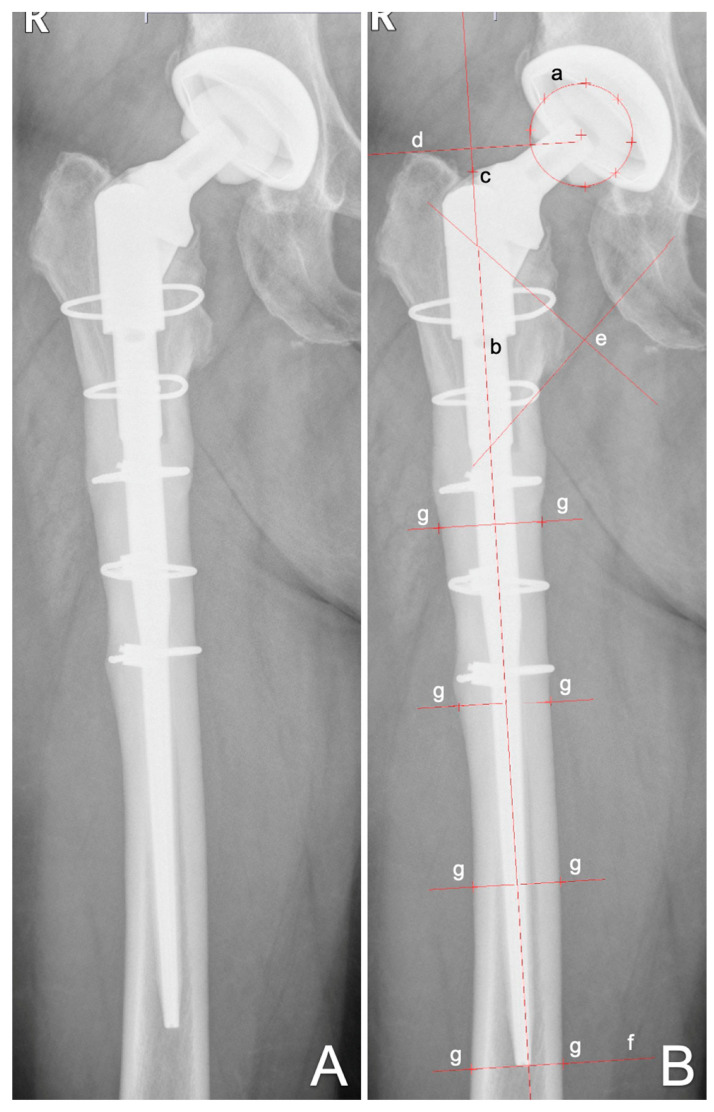
Anterior to posterior X-rays showing a cementless MP stem (**A**) and with EBRA-FCA references (**B**): (a) head points; (b) stem axis; (c) stem shoulder; (d) major trochanter line; (e) minor trochanter lines; (f) tip-of-stem line; (g) points at femoral bone contour.

Axial stem migration, prosthetic stability as well as tilting of the stem were assessed retrospectively with EBRA-FCA from plain X-rays [12,16]. A total of 19 reference points are defined on the femoral head (n = 7), the stem (n = 2), the femoral cortex (n = 8), and one each at the major and minor trochanter [12]. Based on these points as well as the distances between them, a computer file is generated that contains all the essential information for analysis [12]. The EBRA-FCA program then calculates the subsidence, the medial and lateral distances between the prosthesis and the bone margins, the angle between bone and stem as well as the size ratio in relation of head size and stem length [12]. The EBRA-FCA software furthermore excludes radiographs with a comparability algorithm, which identifies significant positioning artifacts by comparing specific bone and prosthetic landmarks. Figure 1 shows the X-ray of an MP stem including EBRA-FCA references.

In our department, we usually follow patients with radiographs before discharge, about 6 weeks after surgery, 12 months postoperative and then in a 1-to-2 year interval. We perform additional radiographs if the patient has any complaints with the THA. All radiographs were taken at our Department of Radiology with the same technique (AP radiographs; patient standing in upright position and full weight-bearing). Migration analysis was performed with EBRA by one independent investigator, who was not involved in the surgeries or postoperative treatment of patients. We defined a minimum of three radiographs per patient, a minimum of three months of radiological follow-up as well as the cementless implantation as inclusion criteria. Accordingly, all patients who did not meet these criteria were excluded.

### Statistics

Mean, median, range, and standard deviation were calculated for the different measurement parameters. For the analysis, Excel (Microsoft Office Professional Plus 2010, Redmond, WA, USA) as well as Graph Pad Prism (Version 8.0, GraphPad Software, Inc., La Jolla, CA, USA) were used. All data was tested for normality using the Kolmogorov–Smirnov Test. For comparison of the EBRA-FCA measurements at different time steps, the Kruskal–Wallis test was used. Comparing the EBRA measurements by BMI and radiolucent lines the Mann–Whitney U-Test was used. When comparing the range of motion pre- and postoperatively, the Mann–Whitney U-Test was used for comparison. A *p*-value of 0.05 was considered statistically significant.

## 3. Results

A total of 67 stems in 62 patients (female: 26; male: 36) fulfilled our inclusion criteria. Patients had a mean age of 68.0 (range 38.7–88.4) years and mean body mass index of 28 (range 19–47) kg/m^2^ at the surgery. Mean follow-up was 33 (range 3–160) months. The three most common preoperative diagnoses were periprosthetic fracture in 22 (32.4%) hips, periprosthetic infection in 19 (27.9%) hips and aseptic loosening in 17 (25.0%) hips. The mean cut-to-suture time was 189 (range 78–428) min. Surgeries were performed in a supine position in 61 (91.0%) and in lateral position in 6 (9.0%) hips. The most commonly used approaches were the direct anterior approach [17] in 25 (37.3%) hips and the lateral approach [18] in 19 (28.4%) hips. Except for the use of femoral strut allografts in two cases, no other form of allografts have been used. More details on patients’ demographics and surgical procedure are shown in Table 1, and details of the implanted stems and cups are shown in Table 2.

EBRA-FCA analysis at 24 months follow-up was calculated for 40 of the 67 stems with an EBRA-FCA-given comparability limit of 3.0 mm (95% confidence interval). On average, 7.0 (range, 3–18) X-rays per implant were analyzed. None of our patients had to be excluded from EBRA-FCA migration analysis. A complete set of radiographs at every single time step (e.g., three, six months, 12 months, etc.) was not available for each stem in our study. Therefore, total subsidence could not be calculated for all cases. This gives a different number of cases in the corresponding migration behavior analysis over time.

**Figure 2:**
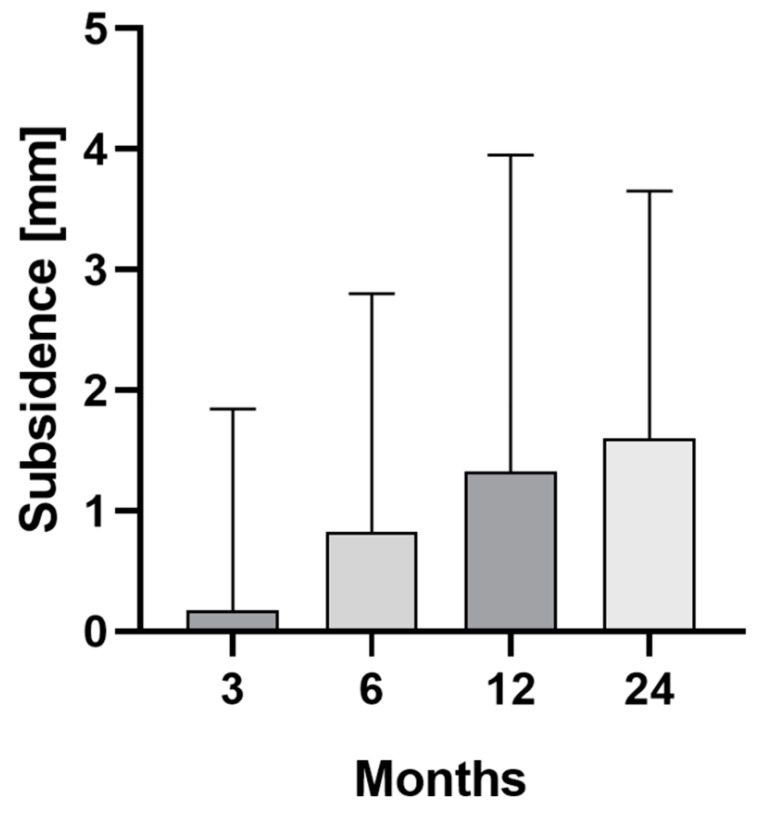
Median and interquartile range (bars) of total stem subsidence for the clinical follow-up of 24 months.

The EBRA-FCA analysis showed a median migration of 0.2 mm (range 0.0–20.6) at three months, 0.8 mm (range 0.0–8.7) at 6 months, 1.3 mm (range 0.0–9.2) at 12 months, and 1.6 mm (range 0.0–7.4) at 24 months after surgery. Thus, the main axial subsidence occurred particularly in the first 6 months postoperatively (Figure 2). The calculated mean monthly axial implant migration was 0.07 mm within the first three months, 0.20 mm between three and six months, 0.08 mm between six and 12 months and 0.03 mm between 12 and 24 months after surgery. No statistically significant difference could be found when comparing the subsidence of implants over time (*p* = 0.059), although Figure 1 shows a tendency toward increasing migration of the stem.

**Figure 3:**
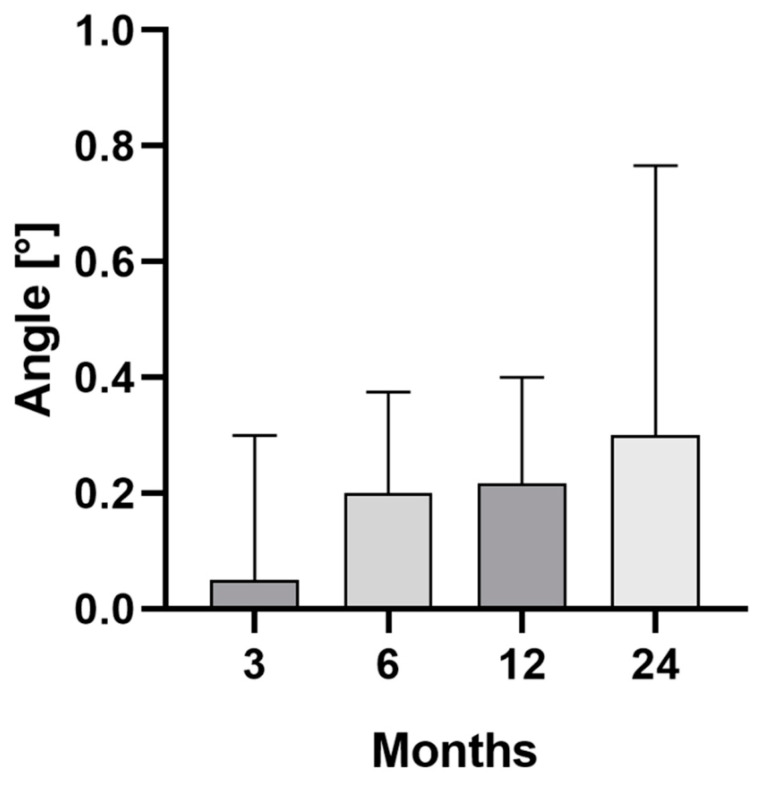
Median and interquartile range (bars) of the angle between stem and femur axis for the clinical follow-up of 24 months.

Additionally, the median angle between stem and femoral axis measured 0.1° (range 0.0°–1.7°) at 3 months, 0.2° (range 0.0°–2.9°) at 6 months, 0.2° (range 0.0°–0.8°) at 12 months, and 0.3° (range 0.1°–2.8°) at 24 months. A statistically significant difference could be found when comparing the angle variation of the stem over time (*p* = 0.009). After 24 months, a statistically significant higher angle could be measured in comparison to 6 months (*p* = 0.005) (Figure 3).

The final radiograph of each patient in our study group was checked for radiolucent areas according to the Gruen zones. Thereby, 23 patients (57.5%) showed a radiolucent margin in at least one of the Gruen zones. This was most frequently seen in Gruen Zones 1 (50%) and 7 (35%). According to the Gruen zones, we divided our patient cohort into two groups to measure its effect on subsidence: patients with radiolucent lines in Gruen zones or not. There was no statistically significant difference in the total migration between the two sub-cohort at 3 months (*p* = 0.0607), at 6 months (*p* = 0.1702), at 1 year (*p* = 0.8925) as well as at 2 years (*p* = 0.2607) follow-up.

Additionally, the patients were divided into groups according to their BMI: normal (BMI  ≤  25 kg/m^2^), overweight (BMI 25.1–29.9 kg/m^2^) and obese (BMI  ≥  30 kg/m^2^). No statistically significant difference could be found between the 3 groups at 3 months (*p* = 0.329), at 6 months (*p* = 0.228), at 1 year (*p* = 0.504) and at 2 years (*p* = 0.269) follow-up. 

A total of five stems (7.5%) from the entire cohort required revision, of which four were due to periprosthetic infection and only one to aseptic loosening. This results in an overall revision-free rate of 92.5% and a revision-free rate for aseptic loosening of 98.5%. Furthermore, no case of material breakage was detected. An intraoperative fracture occurred in three cases (4.5%).

Pre- and postoperative comparison of the range of motion showed a statistically significant mean improvement in flexion by 6° (range, −25°–40°, *p* = 0.018) and internal rotation by 4° (range, −15°–20°, *p* = 0.024). No statistically significant difference could be found between pre- and postoperative evaluation for the external rotation (mean difference 3°, range, −10°–25°, *p* = 0.136), abduction (mean difference 3°, range, −10°–20°, *p* = 0.604) and adduction (mean difference 2°, range, −15°–30°, *p* = 0.156). Preoperatively, a flexion ≥ 90° was possible in 65% of the investigated hip joints, which increased to 94% postoperatively.

## 4. Discussion

Revisions in THA in the presence of a proximal femoral bony defect are a major challenge that can be addressed by using a distally fixed modular stem [19]. In this study, we analyzed the clinical outcome as well as the subsidence of the modular MP Stem by Link (Waldemar Link, Hamburg, Germany). To the best of our knowledge, this is the first study to investigate the migration behavior of the MP stem using EBRA-FCA. At 24 months, we can report a low median subsidence of 1.6 mm as well as a low median axial deviation of 0.3 degrees between the stem and femoral axis. Furthermore, the overall revision-free rate in our study cohort amounted to 92.5%.

Subsidence of the stem has shown to be a negative predictive sign for successful osseointegration and was also considered to be one of the most common risk factors for re-revision, whereby the importance and extent of subsidence varies by implant design [16,20,21]. Furthermore, the reason for revision can also be associated with an increased risk of subsidence. Especially after revision surgery due to periprosthetic fractures, subsidence is frequently observed [22]. With a specificity of 100% and a sensitivity of 78% for detection of migration of more than 1 mm, as compared with RSA, EBRA-FCA is suitable for identifying and measuring the subsidence of femoral components in THA [12]. While RSA is deemed to be the gold standard for migration measurement, the perioperative implantation of tantalum marker balls, which are inserted into the greater and lesser trochanters as well as distal to the tip of the stem, is a prerequisite for RSA. For the subsequent migration measurement, two X-ray images from different angles are required, which are taken with the use of a special calibration cage. The use of special software subsequently enables the three-dimensional evaluation of the X-ray images and the determination of the migration of the implant. EBRA-FCA offers the advantage of being a non-invasive method that can be used in our retrospective study design [23].

Numerous other studies have reported subsidence rates for the MP stem using measurement methods other than EBRA-FCA. While Kwong et al. demonstrated a mean reduction of 2.1 mm (range 0–11.3 mm) at a mean follow-up of 40 months, Houdek et al. presented a mean reduction of 4 mm (range 0–22 mm) at a mean follow-up of 62 months [19,24]. In addition, the median vertical stem migration in the study by Weiss et al. was up to 2.7 mm (range 0–33 mm) at a median follow-up of 6 years [25]. Other modular stems were part of radiological studies. Thus, subsidence for the Modular Restoration Stem system (Stryker, Kalamazoo, Michigan, USA) was 2.5 mm (range 2–29) after 2.4 years, according to Patel et al. [26]. When investigating the same stem, Jayasinghe et al. reported an axial migration of 4.18 after 4.3 years [27]. For the Revitan stem (Zimmer Biomet, Warsaw, Indiana, USA), migration analysis by Girard et al. showed an average subsidence of 3 mm after 5.9 years [20]. In the present study, we can show a median subsidence of 1.6 mm at 24 months for the cementless MP stem. In contrast to other studies, we were able to describe the migration behavior over time. Here, the largest monthly migration of 0.2 mm was documented between 3 and 6 months, decreasing to 0.03 mm between 12 and 24 months. This migration behavior is in close agreement with numerous other reports showing that the largest migration of cementless stems occurs within the first 12 months and results in secondary stabilization [28].

In addition to the subsidence, the appearance of radiolucent lines is repeatedly used as a criterion for stem stability [29]. In order to be able to describe the occurrence in relation to the prosthesis, Gruen et al. divided the cementless hip prosthesis in 7 zones on the ap X-ray of the femoral component [15]. Schuh et al. examined the MRP titanium revision stem (Peter Brehm Chirurgie Mechanik, Weisendorf, Germany) and found radiolucent lines in 34 of the 79 implants (43.0%) at a mean follow-up of 4 years, although these were limited to zones 1 and 7 and only occurred in stems with a diameter of 18 mm or more [30]. After a median follow-up of 11 years, 10.2% of the investigated ZMR modular, cementless femoral component showed radiolucent lines in Gruen zone 1 and 7 [31]. In a study by Stimac et al., 19% of the stems of the Modular Restoration Stem system by Stryker showed radiolucent lines in zones 1, 2, 3 and 7 after a mean follow-up of 4.3 years [32]. In the present study, 53.5% of MP stems had radiolucent lines after 2 years, with 85% projecting to zones 1 and 7. However, after two years, there was no statistically significant difference (*p* = 0.2607) in the migration of the stems with and without radiolucent lines. While radiolucent lines appeared mainly in zones 1 and 7, it can be concluded that the distal anchorage mechanism of the MP stem allows a sufficient fit, resulting in no observed revision for aseptic loosening during our follow-up.

While the modularity of these stem types allows the surgeon to address the bony situation as individually as possible, there are repeated reports of mechanical implant failure, with the connection between the neck and the stem being a particular point of weakness [33,34]. Reported predisposing factors include patient-related factors such as increased body weight and high activity levels, as well as surgery-related factors including a small medullary channel (resulting in a small stem diameter) and deficient proximal bone support, particularly medially [34,35,36,37]. Lakstein et al. studied six fractures of a cementless modular revision stem (four ZMR XL stems and two ZMR stems used with allografts, Zimmer, Warsaw, Indiana), with all stems fracturing between 13 and 80 months after implantation 1 to 2 mm proximal to the body-stem junction [35]. All patients with fractures had significantly increased weight and radiologically showed insufficient bony support around the modular junction [35]. Next to this, Efe et al. reported four revisions of fractured modular stems, whereof three were cementless distally fixed stems (two ZMR standard stems and one MP stem) [33]. Additionally, in these cases, all patients had an increased BMI and showed radiological signs of loosening of the proximal component [33]. The authors pointed out that in addition to good distal fixation, adequate medial proximal bone is required to avoid failure of the femoral component under all loading conditions [33]. In our study, we could not observe any case of material breakage, which precluded an analysis of the influence of BMI. However, increased BMI had no significant influence on subsidence.

The current study has some limitations. First, this study has a retrospective study design, which resulted in the exclusion of some of the treated patients from the study. This potentially makes the study more susceptible to selection bias. Second, the study focused on one specific stem type, which did not allow for a comparison of the outcome with alternative stem types. Third, the varying number of radiographs of each hip during the follow-up, in combination with the smoothing function of EBRA-FCA, may have influenced migration results. Fourth, we were not able to evaluate the clinical outcome using a specific hip score.

## 5. Conclusions

In summary, the MP reconstruction prosthesis showed low median subsidence as well as a reduction in the migration rate over the investigated follow-up. Furthermore, no mechanical failure has been reported. Based on this, the modular stem can be considered as a good therapy option in challenging stem revisions offering various options to address the individual anatomical situation. However, further studies with a larger patient cohort and longer observation period are needed to confirm the results.

## Figures and Tables

**Table 1:** Patients demographics of the study group. Range is given in brackets.

Number of patients	Female	26
	Male	36
	Total	62
Mean age (years)		68 (39–89)
BMI (kg/m^2^)		28 (19–47)
Cut-to-suture time (min)		189 (78–428)
Surgical approach	Direct anterior approach	25
	Extended direct anterior approach	11
	Tensor release	5
	Lateral approach	19
	Posterior approach	6
	Anterolateral approach	1
Surgical position	Supine	61
	Lateral	6
Preoperative diagnosis	Periprosthetic fracture	22
	Periprosthetic infection	19
	Aseptic loosening	17
	Hip dysplasia	2
	Femoral neck fracture	2
	Material breakage	1
	Failed osteosynthesis	1
	Pathologic fracture	1
	Osteoarthritis	1
	Recurrent dislocation	1
Vancouver classification	Type B2	20
	Type B3	2

**Table 2:** Details of implanted components.

Stem product	MP	67 (100)
Anchoring method	Cementless	67 (100)
CCD angle	126°	45 (67)
	135°	12 (18)
	n.a.	10 (15)
Neck length (mm)	35	40 (60)
	65	17 (25)
	n.a.	10 (15)
Stem length (mm)	180	14 (21)
	210	28 (42)
	250	13 (19)
	290	3 (4)
	330	1 (2)
	n.a.	8 (12)
Head size (mm)	28	40 (60)
	32	15 (22)
	36	3 (5)
	n.a.	9 (13)
Head material	Biolox	41 (61)
	CoCrMo	15 (22)
	n.a.	11 (17)

n.a., not assessed.

## Data Availability

Data available on request due to restrictions, e.g., privacy or ethical. The data presented in this study are available on request from the corresponding author.
